# Neural encoding of auditory processing in schizophrenia

**DOI:** 10.3389/fnins.2026.1893300

**Published:** 2026-09-15

**Authors:** Lakshma Reddy Vuyyuru, Anitha Raju

**Affiliations:** Department of Computer Science and Engineering, Koneru Lakshmaiah Education Foundation, Vaddeswaram, Andhra Pradesh, India

**Keywords:** auditory cortex, brain encoding, fMRI, language, schizophrenia, speech models, voxel-wise models

## Abstract

**Introduction:**

Auditory processing abnormalities are a core feature of schizophrenia and are linked to deficits in speech perception, cognitive function, and everyday communication. It remains unclear which stages of auditory processing are most disrupted and how these disruptions relate to symptoms at the level of population-wide neural representations.

**Methods:**

We used fMRI recordings acquired while individuals with schizophrenia and matched healthy controls completed a controlled block-design speech-perception task. Voxel-wise encoding models mapped self-supervised speech-model representations, together with complementary acoustic and linguistic features, to neural responses.

**Results:**

Across cross-validated evaluations, individuals with schizophrenia showed numerically higher encoding alignment than matched controls across all evaluated regions of interest, although no effect survived false discovery rate correction in this small sample. The largest numerical group differences occurred in frontal language regions, including the middle frontal gyrus and dorsomedial prefrontal cortex, and in early auditory cortex. Subgroup analyses suggested that patients without auditory verbal hallucinations drove much of this numerical separation.

**Discussion:**

These descriptive, hypothesis-generating findings suggest that model-based encoding can reveal a frontal-auditory signature in schizophrenia, but they do not establish a clinically actionable biomarker or preserved or enhanced speech encoding. Larger, adequately powered studies with richer feature spaces are needed for confirmatory inference and to test whether encoding-based measures can serve as clinically meaningful biomarkers.

## 1 Introduction

Understanding how the human brain represents and processes complex stimuli is a central goal in both neuroscience and artificial intelligence. Brain encoding models provide a principled framework for studying this question by mapping representations derived from computational models to neural activity, typically measured using functional magnetic resonance imaging (fMRI) (Mitchell et al., 2008; Naselaris et al., 2011; Wehbe et al., 2014; Huth et al., 2016; Jain and Huth, 2018). In this framework, stimulus features extracted by a computational model are linearly projected onto voxelwise BOLD responses, and the variance explained on held-out data quantifies how well the model captures the representational structure of the brain. Early work using hand-engineered acoustic, phonetic, and semantic features established that voxelwise encoding can recover detailed cortical maps of speech and language processing (Huth et al., 2016; de Heer et al., 2017; LeBel et al., 2021; Caucheteux and King, 2022; Vaidya et al., 2022; Tuckute et al., 2023).

This raises an important question: can brain-encoding approaches quantify systematic differences in how computational stimulus representations align with neural activity in individuals with schizophrenia and healthy controls? Encoding models complement classical contrast-based fMRI analyses (Soler-Vidal et al., 2022) by evaluating how well acoustic, speech-model, and linguistic representations predict held-out neural responses. In the present study, feature-family analyses assess the unique predictive contributions of these representational levels across the pooled sample, whereas the clinical group comparisons provide exploratory tests of whether overall encoding alignment differs between HC and SCZ and between AVH+ and AVH− participants. The latter comparisons are not treated as confirmatory evidence that a particular representational stage is disrupted in schizophrenia.

More recently, large-scale neural network models, particularly transformer-based language models (Devlin et al., 2019) and self-supervised speech model (Baevski et al., 2020; Radford et al., 2023), have been shown to predict brain responses to naturalistic stimuli with substantially higher accuracy than classical features (Toneva and Wehbe, 2019; Schrimpf et al., 2021; Caucheteux and King, 2022; Goldstein et al., 2022; Antonello et al., 2021; Oota et al., 2023). Internal representations from models such as GPT-2 (Radford et al., 2019), BERT (Devlin et al., 2019), wav2vec 2.0 (Baevski et al., 2020), and HuBERT (Hsu et al., 2021) align hierarchically with the cortical hierarchy of language processing, with low- and middle-layer representations best predicting auditory cortex and higher layers best predicting associative language regions (Jain and Huth, 2018; Toneva and Wehbe, 2019; Vaidya et al., 2022; Millet et al., 2022; Caucheteux and King, 2022; Oota et al., 2023). These convergences suggest that modern self-supervised models capture aspects of the representational geometry underlying human auditory and linguistic cognition, and that encoding-model performance, often called brain alignment, can serve as a quantitative readout of how a given representational level is instantiated in cortex.

Despite this progress, nearly all encoding-model work has been conducted in neurotypical participants performing naturalistic listening or reading tasks (Schrimpf et al., 2021; Goldstein et al., 2022; LeBel et al., 2021). Far less is known about how model-brain alignment behaves in clinical populations, where the underlying neural representations may be systematically altered. This is a meaningful gap: if encoding models track the representational content of cortex, then disorders that disrupt sensory or linguistic representations should manifest as measurable, regionally specific changes in alignment. Schizophrenia is a particularly compelling test case, because it is associated with well-documented disruptions of auditory perception, speech processing, and semantic integration (Javitt and Sweet, 2015; Kuperberg, 2010), and because these disruptions are thought to span multiple stages of the auditory-to-linguistic hierarchy.

At the sensory end of this hierarchy, schizophrenia patients show reduced activation in primary auditory cortex during passive speech listening, consistent with an early information-processing deficit (Soler-Vidal et al., 2022; Javitt and Sweet, 2015). Post-mortem and structural studies further report neuronal pathology in primary and secondary auditory regions (Sweet et al., 2009), and clinical high-risk individuals exhibit impaired theta-band cortical tracking of the speech envelope (Dondé et al., 2024, 2025). At higher levels, semantic processing is altered: meta-analyses of the N400 component reveal both abnormal automatic spreading activation and impaired late contextual integration in patients (Wang et al., 2011; Kiang and Gerritsen, 2019). One of the most prominent symptoms, auditory verbal hallucinations (AVHs), is associated with atypical activation and connectivity patterns in temporal and frontal language regions, including the superior temporal gyrus (STG), middle temporal gyrus, and inferior frontal gyrus (IFG) (Jardri et al., 2011; Kühn and Gallinat, 2012; Ćurčić-Blake et al., 2017). These findings have been integrated within predictive-coding accounts that frame psychosis as an imbalance in the precision of hierarchical predictions and prediction errors (Adams et al., 2013; Sterzer et al., 2018; Corlett et al., 2019), suggesting that group differences should appear not only as overall hypoactivation, but as a shift in the relative weight of representations across the cortical hierarchy.

The controlled speech-perception paradigm provides a useful testbed because it engages auditory and language systems implicated in schizophrenia while manipulating intelligibility and linguistic content through white noise, reversed speech, words, and sentences (Soler-Vidal et al., 2022). However, unlike the continuous naturalistic-listening paradigms in which modern speech and language models have most often been validated as brain predictors (Vaidya et al., 2022; Antonello et al., 2024), the present experiment uses a block design with relatively limited contextual variation. We therefore compare the deep-feature encoding model against block timing, sound-vs.-silence, acoustic, duration, intelligibility, condition-label, and simple lexical baselines to determine whether the model-derived representations improve held-out neural prediction beyond the structure of the task.

In this work, we apply brain encoding methods to an fMRI speech-perception dataset comprising schizophrenia patients with and without auditory hallucinations, as well as matched healthy controls (Soler-Vidal et al., 2022). The complete experimental workflow is illustrated in Figure 1. Following the encoding-model tradition, we extract a multi-level feature hierarchy from each stimulus, low-level acoustic descriptors (log-mel spectrograms, MFCCs), intermediate self-supervised speech representations from wav2vec 2.0 and HuBERT (Baevski et al., 2020; Hsu et al., 2021; Vaidya et al., 2022; Millet et al., 2022), and high-level contextual embeddings from transformer language models (Devlin et al., 2019), and fit voxelwise banded ridge regression models that predict BOLD activity from these features (Nunez-Elizalde et al., 2019; La Tour et al., 2022). By systematically comparing encoding performance across groups, regions, stimulus conditions, and feature families, we aim to characterize whether and where model–brain alignment captures clinically relevant differences in neural representation, and whether model–brain alignment differs by hallucination status.

**Figure 1:**
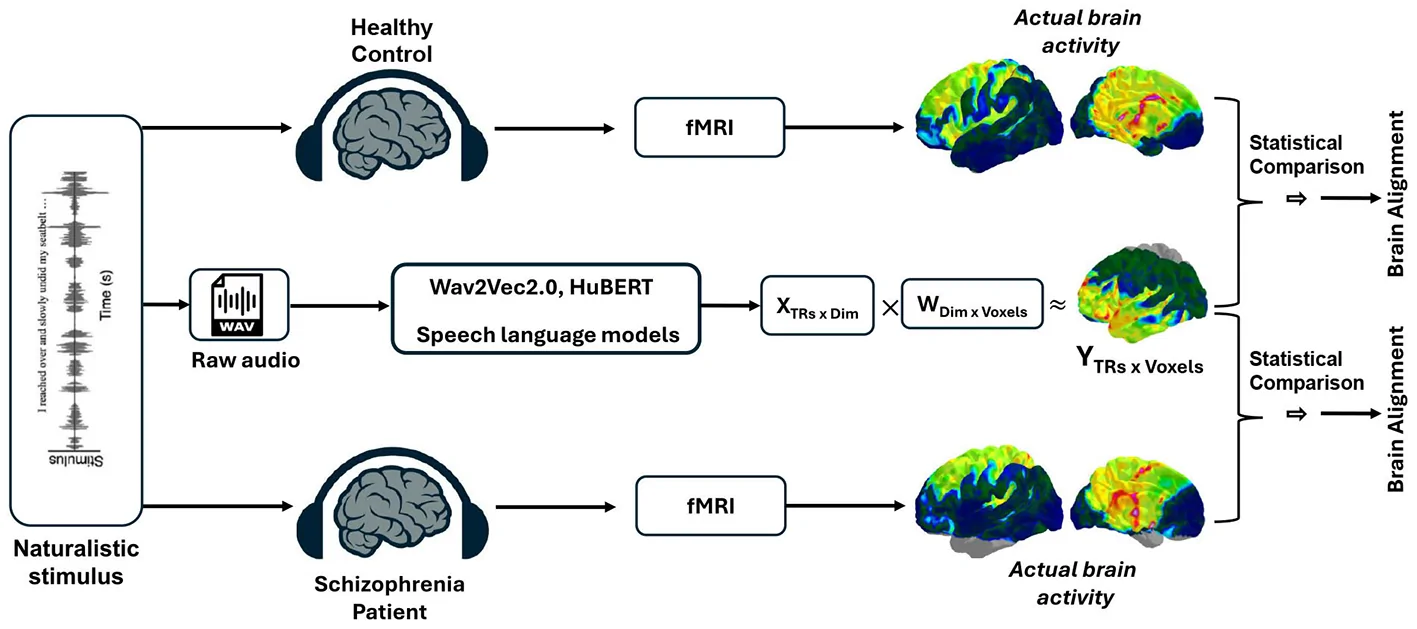
Overview of the experimental workflow for comparing speech-language-model representations with cortical responses in healthy and schizophrenia patients. The controlled block-design speech-perception stimuli is presented along three parallel pathways. In the top pathway, healthy participants listen to the stimulus while undergoing fMRI, yielding voxel-wise blood-oxygen-level-dependent (BOLD) responses (actual brain activity). In the middle pathway, the raw audio waveform is fed into self-supervised speech language models (Wav2Vec 2.0, HuBERT) to extract layerwise embeddings X (TRs × Dim), TR, repetition time; a ridge-regression encoding model then learns a weight matrix W (Dim × Voxels) that maps these embeddings onto brain activity, producing predicted responses Y (TRs × Voxels). In the bottom pathway, schizophrenia patients listen to the same stimulus during fMRI to obtain their voxelwise responses. The model-predicted activity Y is compared against the measured activity in each group, and the resulting brain alignment scores (encoding-model performance) are tested for significant differences between healthy and schizophrenia cohorts, revealing where speech-driven neural representations diverge in schizophrenia. TR, repetition time; dim, feature dimension.

### 1.1 Research questions

We investigate the following research questions to understand the relationship between model representations and brain activity in clinical populations:

- RQ1 (Group Differences): Do brain encoding models exhibit different levels of alignment with neural activity in schizophrenia patients compared to healthy controls, particularly in auditory and language-related regions?
- RQ2 (Hallucination Effects): Are differences in model-brain alignment modulated by the presence of auditory verbal hallucinations (AVH+ vs. AVH-), suggesting distinct representational alterations?
- RQ3 (Stimulus Dependence): Does model-brain alignment vary across stimulus conditions (words, sentences, unintelligible/reversed speech, baseline), and does any such variation differ across groups, or is it primarily driven by block-level stimulus presence rather than linguistic content?
- RQ4 (Regional Specificity): Which brain regions (e.g., Early Auditory cortex, MFG, IFG, IFGOrb, dmPFC, PTL, AG) show the strongest and most differential alignment patterns, and do these reflect known functional disruptions in schizophrenia?

Across cross-validated voxelwise encoding, schizophrenia patients showed numerically higher model–brain alignment than matched controls across all evaluated ROIs, though no effect survived false discovery rate (FDR) correction; the effects were small and the direction (schizophrenia group (SCZ)> healthy controls (HC)) is counterintuitive for a stimulus-locked speech-encoding model. The largest group differences were concentrated in frontal language regions (MFG, dmPFC) and early auditory cortex rather than being uniformly distributed (RQ1, RQ4). Within the patient group, AVH- showed numerically higher encoding alignment than AVH+ in most ROIs, although no subgroup comparison survived corrected inference (RQ2). Condition-wise analyses revealed group-dependent stimulus effects, but the current feature space appears to capture block-level stimulus presence more than linguistic content (RQ3). We therefore present the clinical group results as descriptive and hypothesis-generating and motivate replication with larger, adequately powered samples and corrected feature-family-specific group comparisons.

Looking ahead, this brain enoding framework points toward an IoT-enabled pipeline: lightweight wearable EEG or audio-response sensors at the edge, streaming to networked infrastructure where encoding-style models score auditory–linguistic alignment over time.

## 2 Related work

This work sits at the intersection of three active research threads: (i) voxelwise brain encoding models for naturalistic language and speech, (ii) the use of deep neural networks, particularly self-supervised speech and language models, as candidate models of auditory and linguistic cortex, and (iii) neuroimaging studies of auditory and linguistic disruption in schizophrenia, including auditory verbal hallucinations. We summarize each below and then position our contribution.

### 2.1 Voxelwise brain encoding models with naturalistic stimuli

Voxelwise encoding models map stimulus features to BOLD activity at the level of individual voxels and are evaluated by their ability to predict held-out responses (Naselaris et al., 2011). The approach was popularized in vision (Kay et al., 2008) and extended to language with the seminal demonstration that distributional semantic vectors predict noun-evoked activity (Mitchell et al., 2008). Subsequent work using long, naturalistic narrative stimuli has produced detailed cortical maps of acoustic, articulatory, and semantic representations across the temporal and frontal lobes (Huth et al., 2016; de Heer et al., 2017), and large-scale public datasets such as the Moth-Radio-Hour fMRI corpus now make multi-hour story-listening data broadly available (LeBel et al., 2021). Methodologically, ridge regression remains the workhorse estimator, with banded ridge regression (Nunez-Elizalde et al., 2019; La Tour et al., 2022) extending the framework to multi-feature spaces by allowing per-feature regularization and principled variance partitioning. To our knowledge, this rich naturalistic-encoding tradition has been applied almost exclusively to neurotypical adults; clinical-population encoding work remains rare.

### 2.2 Deep speech and language models as models of auditory and linguistic cortex

A rapidly growing literature shows that intermediate representations of pretrained deep networks are strong predictors of brain responses to speech and language. On the linguistic side, Schrimpf et al. (2021) found that representations from autoregressive transformer language models such as GPT-2 predict fMRI and ECoG responses across the human language network, and that brain-predictivity tracks next-word prediction performance. Goldstein et al. (2022) reported convergent evidence in intracranial recordings, framing prediction-in-context as a shared computational principle between humans and large language models. Caucheteux and King (2022) extended this picture to MEG and fMRI during story listening, providing evidence for a long-range, hierarchical predictive-coding scheme in which higher-order cortical regions encode predictions further into the future. Toneva and Wehbe (2019) and Antonello et al. (2024) further showed that model–brain alignment scales with model size and depth, while Tuckute et al. (2024) demonstrated that GPT-based encoding models are accurate enough to causally drive and suppress responses in the language network via model-selected stimuli.

On the auditory side, self-supervised speech models, APC, wav2vec, wav2vec 2.0 (Baevski et al., 2020), and HuBERT (Hsu et al., 2021), have likewise emerged as strong cortical predictors. Vaidya et al. (2022) systematically benchmarked these models against acoustic baselines and supervised audio networks and found that middle-layer SSL representations yield state-of-the-art prediction performance for fMRI in primary and secondary auditory cortex. Millet et al. (2022) reported convergent results, showing that wav2vec 2.0 representations recapitulate the cortical hierarchy from early auditory regions to higher-level linguistic areas, and that the model's own layers exhibit a similar early-acoustic-to-higher-phonological gradient Pasad et al. (2021); Antonello et al. (2024) extended this analysis across model families and scales, including HuBERT, WavLM, and Whisper. Together, this body of work motivates our specific choice of wav2vec 2.0 and HuBERT as intermediate-level feature spaces: they are currently the best-validated computational descriptors of auditory-cortical responses to naturalistic speech and span a range of phonetic-to-sub-lexical abstractions.

### 2.3 Neural bases of auditory and linguistic disruption in schizophrenia

Schizophrenia is associated with disruption at multiple levels of the auditory-to-linguistic hierarchy. At the sensory level, Javitt and Sweet (2015) reviewed converging evidence for impaired tone-matching, mismatch-negativity, and pitch processing, arguing that auditory dysfunction is a core, mechanistically grounded feature of the disorder rather than a downstream consequence of cognitive deficits. Post-mortem work by Sweet et al. (2009) localized reduced dendritic-spine density to auditory cortex in schizophrenia, providing a cellular substrate for these deficits. Functional studies have repeatedly reported attenuated activation in primary auditory cortex during passive speech listening; the dataset we analyze in this work itself shows reduced left Heschl's-gyrus activation during sentences and reversed speech in schizophrenia patients, regardless of hallucination status Soler-Vidal et al. (2022).

At higher levels, semantic and linguistic processing is altered in characteristic ways. Meta-analyses of the N400 ERP component reveal abnormal automatic spreading activation and impaired late contextual integration in patients (Wang et al., 2011), and word-level semantic prediction deficits index symptom severity (Kiang and Gerritsen, 2019; Kuperberg, 2010). MEG/EEG studies of cortical speech tracking find reduced phase-locking to the speech envelope in clinical-high-risk and first-episode populations, suggesting altered low-frequency entrainment that may precede full diagnosis Dondé et al. (2024). These findings collectively suggest that schizophrenia is not characterized by a single locus of disruption but by a graded set of alterations distributed across acoustic, phonetic, and semantic processing stages—an observation that motivates the multi-level feature hierarchy used in our encoding models.

### 2.4 Neuroimaging of auditory verbal hallucinations

Auditory verbal hallucinations (AVHs), reported by roughly 70% of schizophrenia patients (Soler-Vidal et al., 2022), have been studied with two complementary fMRI paradigms: state studies, which contrast periods of active hallucination with silent periods within the same patient, and trait studies, which contrast hallucination-prone (AVH+) and hallucination-free (AVH-) patients during external tasks. Coordinate-based meta-analyses (Jardri et al., 2011; Kühn and Gallinat, 2012) converge on a bilateral fronto-temporal network including the inferior frontal gyrus, superior and middle temporal gyri, insula, and inferior parietal lobule. Trait studies further show reduced left STG/MTG activation in AVH+ patients during external speech, consistent with the hypothesis that endogenously generated activity competes with bottom-up auditory input (Ćurčić-Blake et al., 2017). Theoretical accounts have framed AVHs as a failure of self-monitoring or, more recently, as overly precise top-down priors within a predictive-coding architecture (Adams et al., 2013; Sterzer et al., 2018; Corlett et al., 2019). Importantly for the present work, Soler-Vidal et al. (2022) found no significant univariate activation differences between AVH+ and AVH- patients on the speech-perception task we re-analyze, motivating the use of a more sensitive, model-based readout that may reveal representational differences invisible to standard contrast analyses.

### 2.5 Encoding models in clinical populations and the present work

Despite the maturity of brain encoding methodology and the depth of clinical fMRI work on schizophrenia, the two threads have rarely been combined. Most encoding-model studies use neurotypical participants Schrimpf et al. (2021); Vaidya et al. (2022); LeBel et al. (2021); Antonello et al. (2024), and most clinical fMRI studies of schizophrenia use univariate contrasts, functional connectivity, or seed-based analyses (Soler-Vidal et al., 2022; Ćurčić-Blake et al., 2017). A small but growing body of work has begun to apply encoding-style approaches in psychiatric contexts, e.g., fine-tuning speech models on brain data (Moussa et al., 2025) or using model-derived surprisal as a neural marker in clinical-high-risk populations (Dondé et al., 2024), but, to our knowledge, no prior study has used multi-level (acoustic, speech-model, linguistic) voxelwise encoding to systematically contrast schizophrenia patients (with and without AVHs) against matched controls during a controlled block-design speech-perception task.

The present work fills this gap. We combine the publicly available (Soler-Vidal et al., 2022) speech-perception fMRI dataset with state-of-the-art self-supervised speech and language models, fit voxelwise banded ridge regression encoding models per participant, and quantify normalized brain alignment across cohorts, regions, and stimulus conditions. This design lets us ask not only whether schizophrenia disrupts model–brain alignment, but which representational level (acoustic, phonetic, semantic) is most disrupted, where in cortex, and whether disruption tracks the presence of auditory verbal hallucinations.

## 3 Dataset details

We use the speech perception fMRI dataset reported by Soler-Vidal et al. (2022), which is publicly available. The final sample comprises 71 right-handed participants: 23 schizophrenia patients with frequent auditory verbal hallucinations (AVH+), 23 schizophrenia patients without hallucinations for at least 6 month s (AVH-), and 25 matched healthy controls. fMRI data were acquired on a 3T Philips Ingenia scanner using a T2*-weighted echo-planar imaging sequence (TR = 2 s, TE = 30 ms), yielding 341 volumes per participant; the first five volumes were discarded to allow for signal stabilization, leaving 336 volumes for analysis. During scanning, participants performed an auditory speech perception task including three conditions: spoken words, spoken sentences, and unintelligible reversed speech. Stimuli were presented in a block-design paradigm with six blocks per condition, each lasting 26 s, separated by 2 s inter-block intervals; a white-noise baseline block was presented after every three stimulation blocks. The total task duration was 11 min 22 s. Participants listened through MRI-compatible headphones while maintaining visual fixation on a gray screen.

### 3.1 Pre-processing for encoding

The original study preprocessed these data in FSL/FEAT for a volume-based general linear model (GLM) analysis (Soler-Vidal et al., 2022). Because voxelwise encoding is more sensitive to preprocessing choices and, in combination with the Glasser parcellation, requires surface-projected data, we pre-processed the raw functional images using fMRIPrep 25.2.0 (NiPreps Developers; distributed open-source project with no single manufacturer location) [internally using FSL 6.0.7 (FMRIB Analysis Group, University of Oxford, Oxford, United Kingdom), ANTs 2.5.1 (Penn Image Computing and Science Laboratory, University of Pennsylvania, Philadelphia, PA, USA), and FreeSurfer 7.3.2 (Laboratory for Computational Neuroimaging, Athinoula A. Martinos Center for Biomedical Imaging, Massachusetts General Hospital, Charlestown, MA, USA)], comprising discarding of the first five volumes, motion correction, co-registration, and normalization to MNI space. Following the parameter choices of Soler-Vidal et al. (2022), images were spatially smoothed with a 5 mm FWHM Gaussian filter, and participants exceeding the original motion thresholds (maximum absolute movement >3.0 mm or mean absolute movement >0.3 mm) were excluded. Each MNI-space functional run was then denoised using nilearn.image.clean_img with 14 nuisance regressors: the six rigid-body motion parameters, their six temporal derivatives, and the mean cerebrospinal-fluid and white-matter signals estimated by fMRIPrep, with confound regression, linear detrending, and high-pass filtering (cutoff 1/128 Hz ≈0.0078 Hz; TR = 2 s) applied jointly. The denoised volumes were projected to the fsaverage6 surface using nilearn.surface.vol_to_surf. Because these preprocessing parameters were adapted from a pipeline designed for volume-based GLM analysis rather than optimized for voxelwise encoding, and because encoding accuracy can be sensitive to such choices, the reported model–brain correspondences should be interpreted with this dependency in mind.

### 3.2 Regions of interest

We adopt the ROI analysis using the Glasser atlas projected onto “fsaverage6” space. However, given the auditory nature of the task, we replace visual processing regions with auditory cortex regions, including primary auditory cortex (A1), superior temporal gyrus (STG), and superior temporal sulcus (STS). We retain higher-level language regions (angular gyrus (AG), lateral temporal cortex (ATL and PTL), inferior frontal gyrus (IFG and IFGOrb), middle frontal gyrus (MFG), posterior cingulate cortex (PCC) and dorsomedial prefrontal cortex (dmPFC)) to capture semantic and integrative processing during speech perception (Milton et al., 2021; Desai et al., 2023).

## 4 Methodology

### 4.1 Feature extraction

To characterize the stimulus at multiple representational levels, we extract features spanning low-level acoustic structure, intermediate speech representations, and high-level linguistic semantics. This multi-level design allows us to test whether neural responses in schizophrenia are better explained by sensory, phonological, or semantic representations.

#### 4.1.1 Acoustic features

For each auditory stimulus, we first extract low-level acoustic descriptors directly from the waveform. These include log-mel spectrogram features, which provide a time–frequency representation of the signal and are widely used to model early auditory responses. We compute 80-bin log-mel filterbank energies using short-time analysis windows and align the resulting features to the fMRI sampling grid after temporal aggregation. We additionally extracted 13 MFCCs and their first- and second-order temporal derivatives (39 features), which were concatenated with the 80-bin log-mel features to form the 119-dimensional acoustic feature family; acoustic envelope/energy was evaluated separately in the baseline analysis.

#### 4.1.2 Speech and phonological features

To capture intermediate representations between raw acoustics and sentence-level meaning, we extract features from pretrained speech models. In particular, we use hidden representations from self-supervised speech encoders such as wav2vec 2.0 (Baevski et al., 2020) or HuBERT (Hsu et al., 2021), which have been shown to encode increasingly abstract phonetic and phonological structure across layers. For each stimulus, we pass the audio waveform through the pretrained model and obtain frame-level hidden states from each layer. These features are then temporally pooled or resampled to match the fMRI repetition time (TR), enabling voxel-wise encoding analyses at multiple representational depths.

#### 4.1.3 Linguistic features

To model higher-level lexical and semantic information, we extract text-based embeddings from pretrained language models using the transcript corresponding to each spoken stimulus. For the word and sentence conditions, we obtain contextual embeddings from transformer-based language models [ e.g., BERT (Devlin et al., 2019) or GPT-style (Radford et al., 2019) models], using either the final hidden layer or a layer-wise set of representations. For each stimulus item, token-level embeddings are aggregated into a single feature vector using mean pooling or by selecting the final token representation, depending on the model architecture. These features are intended to capture lexical identity, compositional semantics, and contextual integration beyond low-level acoustics.

#### 4.1.4 Pre-trained model specification

The pretrained checkpoints and principal feature-extraction and temporal-alignment settings are summarized in Table 1. We extracted speech representations using Wav2Vec 2.0 base (facebook/wav2vec2-base) and HuBERT base (facebook/hubert-base-ls960). Both are self-supervised speech encoders with 12 transformer layers and 768-dimensional hidden states. The Wav2Vec 2.0 base checkpoint was pretrained on LibriSpeech audio, whereas facebook/hubert-base-ls960 was pretrained on the 960-h LibriSpeech corpus.

**Table 1:** Pre-trained checkpoints used for computational feature extraction.

Model	Hugging face checkpoint	Architecture	Layers	Hidden size
Wav2Vec 2.0 base	facebook/wav2vec2-base	Speech encoder	12	768
HuBERT base	facebook/hubert-base-ls960	Speech encoder	12	768
BERT base uncased	google-bert/bert-base-uncased	Bidirectional text encoder	12	768
GPT-2 small	openai-community/gpt2	Causal text decoder	12	768

We extracted text representations using BERT base uncased (google-bert/bert-base-uncased) and GPT-2 small (openai-community/gpt2). BERT is a 12-layer, 768-dimensional bidirectional transformer encoder pretrained on English Wikipedia and BookCorpus. GPT-2 small is a 12-layer, 768-dimensional causal transformer decoder with approximately 124 million parameters, pretrained on WebText. Representations were extracted from layer 7. All models were used in evaluation mode with their pretrained parameters frozen; no fine-tuning was performed. Models were publicly available at Hugging Face.

#### 4.1.5 Audio pre-processing and speech-feature extraction

The stimulus audio was converted to mono and resampled to 16 kHz, matching the input sampling rate required by the Wav2Vec 2.0 and HuBERT checkpoints. The waveforms were processed in 16-s context windows advanced in 100-ms increments. Each window therefore represented the acoustic context preceding and including the corresponding evaluation time point. Each audio window was passed through the frozen Wav2Vec 2.0 and HuBERT encoders. We retained the 768-dimensional hidden state from transformer layer 7 at the final valid temporal frame of each window. Here, “final temporal frame” refers to the final speech-encoder output within the window and not to a discrete linguistic token.

#### 4.1.6 Text pre-processing and language-feature extraction

Text representations were obtained from the transcripts corresponding to the presented words and sentences. To extract the text features from these pretrained models, we constrained the tokenizer to use a maximum context of 20 words. BERT inputs were tokenized using the WordPiece tokenizer distributed with google-bert/bert-base-uncased; GPT-2 inputs were tokenized using the byte-level BPE tokenizer distributed with openai-community/gpt2. For each context, we extract the hidden state from layer 7. The context representation is defined using last token representation. Token representations are aligned with stimulus time using temporal alignment with fMRI.

#### 4.1.7 Condition-specific handling

The dataset contains three conditions: spoken words, spoken sentences, and reversed unintelligible speech. For the word and sentence conditions, we extract all three feature families: acoustic, speech-model, and linguistic features. For reversed speech, we extract acoustic and speech-model features from the audio signal, but we do not assign semantic text embeddings, since the reversed stimuli are unintelligible by design. This distinction helps isolate whether model-brain alignment differences arise from semantic content or from lower-level auditory structure.

#### 4.1.8 Temporal alignment with fMRI

All feature sequences are aligned to the fMRI data at the repetition time of 2 s. For time-varying features such as spectrograms or speech-model hidden states, we aggregate feature frames within each TR window using mean pooling. To account for the delayed hemodynamic response, we evaluate both a finite impulse response lag model and a standard delayed-feature approach in which stimulus features are concatenated across several post-stimulus lags. This allows the encoding model to capture the temporal offset between auditory processing and the observed BOLD response.

#### 4.1.9 Dimensionality reduction and normalization

Because pretrained model features can be high dimensional, we apply standardization within each feature family and optionally reduce dimensionality using principal component analysis (PCA), retaining a fixed percentage of explained variance or a fixed number of components. This improves numerical stability and allows fairer comparisons across feature types. All transformations are fit on the training data only and then applied to held-out data.

#### 4.1.10 Baseline-model comparisons

To determine whether encoding performance was attributable to the block-design structure rather than to stimulus representations, we constructed a series of increasingly detailed baseline models. The baseline ladder contained: (1) block timing; (2) sound-vs.-silence; (3) acoustic envelope/energy; (4) stimulus duration; (5) intelligibility; (6) separate indicators for white noise, reversed speech, words, and sentences; and (7) simple lexical controls comprising word count, phoneme count, mean log word frequency, and sentence length.

#### 4.1.11 Feature comparisons

Using this feature hierarchy, we compare three levels of representation: (i) low-level acoustic features that primarily reflect auditory structure, (ii) intermediate speech-model features that capture phonetic and sub-lexical regularities, and (iii) high-level linguistic embeddings that capture lexical and semantic content. Comparing these feature families enables us to test whether group differences in schizophrenia are most prominent at early auditory, speech-perceptual, or semantic representational stages.

## 5 Experimental setup

Table 2 summarizes the complete pipeline used for the main reported results, from feature extraction through group-level statistics. The remainder of this section details the encoding model and evaluation.

**Table 2:** Summary of the encoding pipeline used for the main reported results.

Pipeline component	Setting used for the main reported results
Feature families (bands)	Three bands, one per family: *Acoustic*: 80-bin log-mel + 13 MFCCs with Δ/ΔΔ (39), 119-d, no PCA; *Speech-model*: wav2vec 2.0 base, 768-d → PCA 256; *Linguistic*: BERT-base contextual, 768-d → PCA 256. Bands defined per family via ColumnKernelizer.
Temporal aggregation	Feature frames averaged (mean pooling) within each 2 s TR
Dimensionality reduction	PCA fit on training data, applied to the model-derived bands only: wav2vec 2.0 → 256, BERT → 256; acoustic retained at full 119-d
Feature dimensionality	119 + 256 + 256 = 631 per delay; with 4 FIR delays, 2, 524 total features (vs. 265 training TRs)
Normalization	Per-family standardization (*z*-scoring) of features and BOLD; all transforms fit on training folds only and applied to held-out data
Lag structure	FIR, 4 delays (1–4 TRs, ~2–8 s) per voxel
Estimator	Banded ridge regression: per-family linear kernels (ColumnKernelizer) fed to MultipleKernelRidgeCV, giving band-specific regularization (Nunez-Elizalde et al., 2019; La Tour et al., 2022)
Regularization	Random search, 100 iterations; per-band α sampled over 9 log-spaced values (10^0^–10^4^)
Evaluation	5-fold cross-validation; hyperparameter search nested within each training fold
Reported quantity	Joint (full-model) prediction accuracy; per-band variance decomposition not computed
Voxel selection	Voxels with Pearson *r* ≥ 0.05
Evaluation metric	Pearson correlation (predicted vs. measured BOLD), averaged within each ROI
Group inference	Welch's two-sample *t* on subject-level ROI mean *r* (RQ1–RQ4)
Multiple comparisons	Benjamini–Hochberg FDR, *q* = 0.05, across the 12 valid ROIs within each RQ

### 5.1 Voxelwise encoding model

We train banded ridge regression based voxel-wise encoding models (Nunez-Elizalde et al., 2019; La Tour et al., 2022) to predict the fMRI brain activity associated with the stimulus representations obtained from speech-based language models. We employ z-score thresholding separately for both input stimulus representations and brain recordings for training and test datasets. For each subject, we account for the delay in the hemodynamic response by modeling hemodynamic response function using a finite response filter (FIR) per voxel with 4 temporal delays (TRs) corresponding to ~8 seconds (Huth et al., 2022). Formally, at each time step *t*, we encode the stimuli as $X_{t}\in {\mathbb{R}}^{D}$ and brain region voxels $Y_{t}\in {\mathbb{R}}^{V}$, where *D* denotes the dimension of the concatenation of delayed 4 TRs, and *V* denotes the number of voxels. Overall, with *N* such TRs, we obtain *N* training examples.

### 5.2 Drop-family variance partitioning

To estimate the unique contribution of each representational family, we perform an exploratory drop-family analysis in the original across all subjects. Let *F* = *A* + *W* + *H* + *B* denote the complete model, where *A*, *W*, *H*, and *B* represent the acoustic, wav2vec 2.0, HuBERT, and BERT feature families, respectively. Unique contributions were defined as


$$U_{A}=r_{F}-r_{W+H+B},\;U_{W}=r_{F}-r_{A+H+B},$$

$$U_{H}=r_{F}-r_{A+W+B},\;U_{B}=r_{F}-r_{A+W+H}.$$

The full and reduced models used identical training and held-out folds, and each full-minus-reduced difference was calculated separately for each participant. Unique contributions were tested against zero using two-sided one-sample *t* tests. Benjamini–Hochberg correction was applied across the 12 valid ROIs separately within each feature family.

Regional differentiation is assessed using a two-way repeated-measures analysis with feature family (acoustic, wav2vec 2.0, HuBERT, and BERT) and ROI as within-participant factors. Partial η^2^ is reported as the effect-size measure.

### 5.3 Evaluation metrics

We evaluate our models using Pearson Correlation (PC), which is a standard metric for evaluating brain alignment (Jain and Huth, 2018; Schrimpf et al., 2021; Goldstein et al., 2022). Let TR be the number of time repetitions in the test set. Let $Y={\left\{Y_{i}\right\}}_{i=1}^{TR}$ and $Ŷ={\left\{{Ŷ}_{i}\right\}}_{i=1}^{TR}$ denote the actual and predicted value vectors for a single voxel, respectively. Thus, *Y* and Ŷ ∈ℝ^*TR*^. We use PC to compute the correlation function, *corr*(*Y*, Ŷ). The final measure of a model's performance is obtained by calculating Pearson's correlation between the model's predictions and neural recordings. For calculating *brain alignment*, we select the voxels with Pearson correlation ≥ 0.05.

## 6 Results

We first quantify the unique contributions and regional distributions of the acoustic, speech-model, and linguistic feature families. Then for each research question, we quantify group or condition differences in ROI-wise encoding accuracy and then interpret the resulting regional pattern in relation to speech processing, hallucination status, and schizophrenia-related changes in auditory-language networks. Throughout results, we refer to healthy controls as HC, the pooled schizophrenia group as SCZ, and the hallucinating and non-hallucinating subgroups as AVH+ and AVH-, respectively.

### 6.1 Baseline models and deep-feature increments

The baseline-model comparisons showed that block timing alone accounted for only limited held-out prediction performance [($\bar{r}$=0.0120); Table 3]. Adding sound-vs.-silence information increased mean performance by (Δ*r* = 0.0041), while acoustic envelope/energy produced a larger increment of (Δ*r* = 0.0109). Stimulus duration, condition identity, and the simple lexical controls produced comparatively small mean increments. The intelligibility step produced a mean increment of (Δ*r* = 0.0170), although no individual ROI survived FDR correction for this step.

**Table 3:** Baseline ladder and deep-model increments in held-out encoding accuracy (*N* = 71).

Model step	Mean held-out *r*	Mean gain *Δr*	FDR-significant ROIs
Block timing	0.0120	–	–
+ Sound-vs.-silence	0.0161	+0.0041	SceneVisual (1/12)
+ Acoustic envelope/energy	0.0270	+0.0109	PTL, PCC, SceneVisual (3/12)
+ Stimulus duration	0.0285	+0.0015	0/12
+ Intelligibility	0.0455	+0.0170	0/12
+ Condition labels	0.0435	-0.0020	0/12
+ Simple lexical controls	0.0453	+0.0018	0/12
+ Wav2Vec 2.0	0.0606	+0.0153	PTL, IFG, MFG, MidTemporal, EarlyAuditory (5/12)
+ HuBERT	0.0733	+0.0127	PTL, MFG, IFGOrb, ObjectVisual (4/12)
+ BERT (complete model)	0.0785	+0.0052	0/12

Mean gain denotes the change in held-out Pearson *r* relative to the preceding model. BH-FDR correction was applied across the 12 valid ROIs separately at each model step. Condition labels comprised separate indicators for white noise, reversed speech, words, and sentences. Simple lexical controls comprised word count, phoneme count, mean log word frequency, and sentence length. Sequential increments are conditional on the displayed model order; drop-family comparisons are reported separately in Table 4.

The complete model substantially exceeded the simpler baselines. Relative to block timing, it improved mean held-out prediction by (Δ*r* = 0.0665), with significant improvements in 9 of 12 ROIs. Relative to the task-structure baseline, the mean improvement was (Δ*r* = 0.0500), with significant improvements in 8 of 12 ROIs. Relative to the rich non-deep baseline containing task structure, acoustic information, intelligibility, and lexical controls, the complete model improved prediction by (Δ*r* = 0.0332), with significant improvements in 7 of 12 ROIs. These direct comparisons are summarized in Table 4.

**Table 4:** Direct comparisons between the complete encoding model and simpler baseline models.

Comparison	Mean gain *Δr*	FDR-significant ROIs
Complete model vs. block timing	+0.0665	9/12
Complete model vs. task-structure baseline	+0.0500	8/12
Complete model vs. rich non-deep baseline	+0.0332	7/12
Unique Wav2Vec 2.0 increment	+0.0153	5/12
Unique HuBERT increment	+0.0127	4/12
Unique BERT increment	+0.0052	0/12

Mean gain is the participant-averaged difference in held-out Pearson *r*. BH-FDR correction was applied across the 12 valid ROIs separately for each comparison.

The task-structure baseline contained block timing, sound-vs.-silence, duration, and condition labels. The rich non-deep baseline contained all task-structure, acoustic, intelligibility, and simple lexical controls but no deep-model representations.

The deep-feature contribution was concentrated in the self-supervised speech representations. Wav2Vec 2.0 contributed a mean unique increment of (Δ*r* = 0.0153), surviving FDR correction in PTL, IFG, MFG, MidTemporal, and EarlyAuditory. HuBERT contributed a mean unique increment of (Δ*r* = 0.0127), surviving correction in PTL, MFG, IFGOrb, and ObjectVisual. The BERT increment was smaller ((Δ*r* = 0.0052)) and did not survive correction in any ROI. Thus, the deep-model improvement was not reducible to the measured block, condition, acoustic, intelligibility, or lexical baselines, but the reliable additional information arose from intermediate speech-model representations rather than from BERT.

### 6.2 Exploratory feature-family variance partitioning

Drop-family comparisons revealed significant regional differentiation among the feature families in the full sample (*N* = 71). The feature-family main effect was significant, *F*(3, 210) = 6.93, *p* < 0.001, partial η^2^ = 0.089, as was the feature-family-by-ROI interaction, *F*(33, 2310) = 7.27, *p* < 0.001, partial η^2^ = 0.093. The ROI main effect did not reach significance, *F*(11, 770) = 1.09, *p* = 0.362, partial η^2^ = 0.015. Thus, the magnitude and regional distribution of the unique encoding contributions differed across feature families. ROI-wise unique contributions and the repeated-measures analysis are reported in Tables 5 and 6, respectively.

**Table 5:** Exploratory drop-family variance-partitioning analysis in the full sample (*N* = 71).

	Acoustic		Wav2vec 2.0		HuBERT		BERT	
ROI	Δ*r*_*A*_	*q*_BH_	Δ*r*_*W*_	*q*_BH_	Δ*r*_*H*_	*q*_BH_	Δ*r*_*B*_	*q*_BH_
AG	+0.011	0.169	+0.006	0.259	+0.009	0.060	+0.010	0.283
PTL	**+0.013**	**0.032**	**+0.018**	**0.0002**	**+0.015**	**0.0006**	+0.004	0.283
IFG	+0.007	0.189	**+0.028**	**0.0007**	+0.010	0.090	+0.009	0.283
MFG	+0.014	0.059	**+0.045**	**0.0001**	**+0.030**	**0.002**	+0.012	0.191
IFGOrb	+0.005	0.254	+0.010	0.104	**+0.024**	**0.002**	+0.008	0.283
PCC	**+0.021**	**0.023**	+0.004	0.259	+0.006	0.120	+0.006	0.283
dmPFC	+0.010	0.254	+0.008	0.259	+0.012	0.090	+0.010	0.283
EarlyVisual	+0.012	0.093	+0.010	0.051	+0.006	0.135	+0.001	0.931
ObjectVisual	+0.004	0.411	+0.008	0.103	**+0.018**	**0.0006**	–0.002	0.783
SceneVisual	**+0.018**	**0.023**	+0.004	0.259	+0.003	0.562	+0.000	1.000
MidTemporal	+0.012	0.093	**+0.022**	**0.005**	+0.010	0.127	+0.003	0.749
EarlyAuditory	+0.004	0.411	**+0.020**	**0.006**	+0.009	0.127	+0.001	0.931
Mean Δ*r*	+0.0109		+0.0153		+0.0127		+0.0052	
FDR-significant ROIs	3/12		5/12		4/12		0/12	

Unique contributions were calculated as the difference in held-out prediction accuracy between the full model and the corresponding reduced model. For each feature family, the first column reports the mean unique contribution, Δ*r*, and the second column reports the BH-adjusted *q*_BH_ value. BH-FDR correction was applied across the 12 valid ROIs separately within each feature family. Bold values survived correction at *q*_BH_ < 0.05.

**Table 6:** Repeated-measures analysis of unique feature-family contributions across ROIs.

Effect	Statistic	*p*	Partial η^2^
Feature family	*F*(3, 210) = 6.93	0.001	0.089
ROI	*F*(11, 770) = 1.09	0.362	0.015
Feature family × ROI	*F*(33, 2310) = 7.27	0.001	0.093

Acoustic features produced significant unique contributions in 3 of the 12 valid ROIs: PTL, PCC, and Scene Visual. Wav2vec 2.0 produced significant unique contributions in five ROIs: PTL, IFG, MFG, Mid-Temporal, and Early Auditory. Its largest contribution occurred in MFG (Δ*r* = 0.045, *q*_BH_ = 0.0001). HuBERT produced significant unique contributions in four ROIs: PTL, MFG, IFGOrb, and Object Visual, with its largest contribution in MFG (Δ*r* = 0.030, *q*_BH_ = 0.002).

In contrast, the unique BERT increment did not survive FDR correction in any ROI. Its mean contribution was smaller ($\bar{\Delta r}=0.0052$) than those of wav2vec 2.0 ($\bar{\Delta r}=0.0153$) and HuBERT ($\bar{\Delta r}=0.0127$). These findings provide evidence for feature-family-specific regional differentiation, with the broadest unique contributions arising from intermediate speech-model representations. The results support a partial acoustic-to-intermediate-speech hierarchy but do not establish a reliably encoded BERT-based lexical-semantic level.

### 6.3 RQ1 finding: SCZ shows numerically higher encoding alignment than HC, but the effect is descriptive and not FDR-significant

To test whether speech-encoding alignment differs between healthy controls and patients with schizophrenia, we compare ROI-wise encoding accuracy between the HC group and the pooled SCZ group across language, auditory, and visual control regions.

#### 6.3.1 Results

Across all valid language and sensory ROIs, the schizophrenia group showed numerically higher encoding accuracy than the healthy control group (Table 7; 12/12 valid ROIs had HC−SCZ < 0). The largest numerical group differences appeared in MFG (Δ*r* = −0.0696, 95% CI [−0.1757, 0.0365], *p* = 0.165, *q*_BH_ = 0.504), SceneVisual (Δ*r* = −0.0464, 95% CI [−0.1921, 0.0993], *p* = 0.367, *q*_BH_ = 0.504), dmPFC (Δ*r* = −0.0445, 95% CI [−0.0895, 0.0005], *p* = 0.052, *q*_BH_ = 0.312), and EarlyAuditory (Δ*r* = −0.0433, 95% CI [−0.0766, −0.0100], *p* = 0.021, *q*_BH_ = 0.252). The ROI-wise HC-SCZ comparison is visualized in Figure 2.

**Table 7:** RQ1: HC vs. SCZ (AVH+/AVH− pooled) per-ROI mean encoding accuracy (Pearson *r* over the held-out test set).

ROI	HC	SCZ	Δ*r*	95% CI	*t*	*p*_unc_	*q*_BH_
AG	–0.0168	+0.0184	–0.0352	[−0.1518, +0.0814]	–1.03	0.387	0.504
ATL	–	–	–	–	–	–	–
PTL	+0.0111	+0.0264	–0.0152	[−0.0625, +0.0321]	–0.90	0.420	0.504
IFG	+0.0193	+0.0480	–0.0287	[−0.0788, +0.0214]	–1.60	0.186	0.504
MFG	–0.0032	+0.0664	–0.0696	[−0.1757, +0.0365]	–1.55	0.165	0.504
IFGOrb	+0.0045	+0.0362	–0.0317	[−0.1128, +0.0494]	–0.94	0.381	0.504
PCC	-0.0018	+0.0151	-0.0169	[−0.0616, +0.0278]	-0.90	0.399	0.504
dmPFC	–0.0055	+0.0391	–0.0445	[−0.0895, +0.0005]	–2.39	0.052	0.312
EarlyVisual	+0.0128	+0.0188	-0.0060	[−0.0264, +0.0144]	-0.70	0.507	0.553
ObjectVisual	+0.0058	+0.0126	–0.0068	[−0.0444, +0.0308]	–0.44	0.675	0.675
FaceVisual	–	–	–	–	–	–	–
SceneVisual	–0.0272	+0.0192	–0.0464	[−0.1921, +0.0993]	–1.08	0.367	0.504
MidTemporal	+0.0002	+0.0118	–0.0115	[−0.0349, +0.0119]	–1.30	0.255	0.504
EarlyAuditory	–0.0116	+0.0318	–0.0433	[−0.0766, −0.0100]	–3.40	0.021	0.252

The raw effect size is defined as Δ*r* = HC − SCZ and is reported with a two-sided Welch 95% confidence interval (CI). The *p* values are uncorrected; *q*_BH_ values were obtained using the Benjamini–Hochberg procedure across the 12 valid ROIs. No comparison survived FDR correction at *q*_BH_ < 0.05.

**Figure 2:**
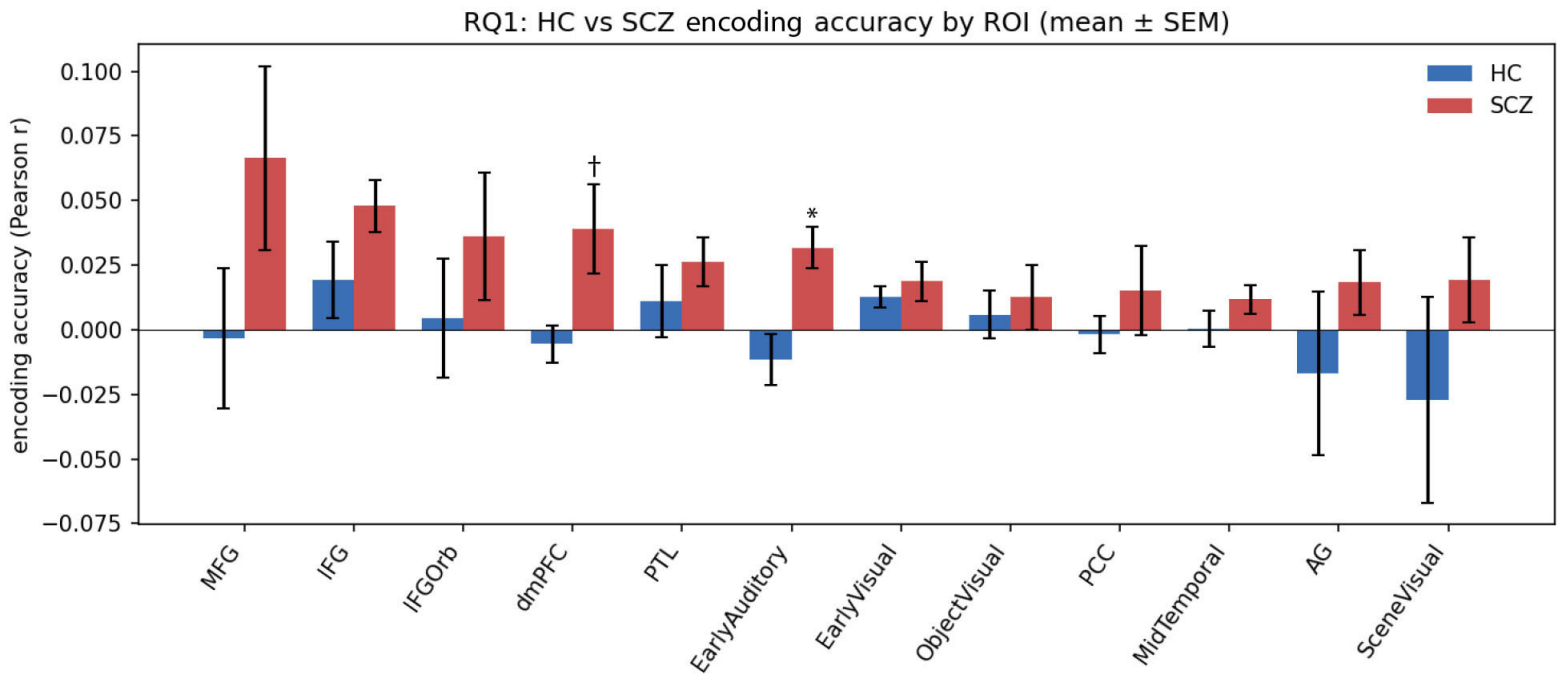
Encoding accuracy by ROI for HC vs. SCZ (mean ± SEM across subjects). ^*^p < 0.05, ^†^*p* < 0.10 (uncorrected, Welch's *t*).

However, this apparent SCZ>HC pattern should be interpreted cautiously. The effects are small in absolute terms (per-ROI *r* differences ≤ 0.07), and no ROI survived Benjamini–Hochberg FDR correction at *q*_BH_ < 0.05. EarlyAuditory was the only ROI whose uncorrected 95% confidence interval excluded zero, but its adjusted value was *q*_BH_ = 0.252. Thus, the group separation was not robust to multiple-comparison control despite its consistent direction across all valid ROIs. The direction is also biologically counterintuitive for a speech-perception encoding model, as one might expect schizophrenia to reduce rather than increase stimulus-locked alignment. An outlier-sensitivity analysis indicated that one low-performing HC subject (sub-03) contributed disproportionately to the HC group mean. After excluding sub-03, the direction of the HC– -SCZ difference was preserved across all 12 valid ROIs. In the key ROIs the difference shrank toward zero, from Δ=-0.043 to Δ=-0.030 in EarlyAuditory, from Δ=-0.045 to Δ=-0.026 in dmPFC, and from Δ=-0.070 to Δ=-0.043 in MFG, and no ROI reached FDR significance after exclusion. The overall interpretation of RQ1 as a consistent-but-unconfirmed numerical separation therefore does not depend materially on this single participant. Overall, the current result should not be taken as evidence that schizophrenia preserves or enhances speech encoding; rather, it indicates a consistent but statistically unconfirmed numerical separation that warrants replication with richer feature spaces and pre-registered, corrected inference.

### 6.4 RQ2 finding: AVH− patients drive the SCZ>HC pattern more than AVH+ patients

To examine whether auditory verbal hallucination status modulates encoding alignment within schizophrenia, we compare ROI-wise encoding accuracy between AVH+ and AVH− patients.

#### 6.4.1 Results

Within the schizophrenia group, AVH+ and AVH− patients did not differ significantly in any ROI (Table 8). All 12 uncorrected 95% confidence intervals included zero, and no comparison survived Benjamini–Hochberg FDR correction at *q*_BH_ < 0.05. Nevertheless, the numerical pattern was consistent across regions: AVH− patients showed higher encoding accuracy than AVH+ patients in 10/12 valid ROIs. The largest subgroup difference occurred in MFG, where AVH− showed higher alignment than AVH+ (AVH+ = 0.0242, AVH− = 0.1085, Δ*r* = AVH+−AVH− = −0.0843, 95% CI [−0.3069, 0.1383], *t* = −1.25, *p* = 0.305, *q*_BH_ = 0.821). Smaller but directionally similar numerical differences appeared in IFGOrb, AG, dmPFC, SceneVisual, and EarlyVisual. The ROI-wise AVH+ versus AVH− comparison is visualized in Figure 3.

**Table 8:** RQ2: AVH+ vs. AVH− per-ROI mean encoding accuracy (Pearson *r* over the held-out test set).

ROI	AVH+	AVH−	Δ*r*	95% CI	*t*	*p*_unc_	*q*_BH_
AG	+0.0043	+0.0324	–0.0281	[−0.0947, +0.0385]	–1.16	0.309	0.821
ATL	–	–	–	–	–	–	–
PTL	+0.0320	+0.0208	+0.0112	[−0.0473, +0.0697]	+0.55	0.614	0.821
IFG	+0.0483	+0.0476	+0.0007	[−0−.0558, +0.0572]	+0.03	0.977	0.977
MFG	+0.0242	+0.1085	–0.0843	[−0.3069, +0.1383]	–1.25	0.305	0.821
IFGOrb	+0.0143	+0.0581	–0.0438	[−0.1906, +0.1030]	–0.87	0.439	0.821
PCC	+0.0061	+0.0241	–0.0181	[−0.1678, +0.1316]	–0.48	0.675	0.821
dmPFC	+0.0266	+0.0516	–0.0250	[−0.1338, +0.0838]	–0.69	0.535	0.821
EarlyVisual	+0.0103	+0.0273	–0.0170	[−0.0582, +0.0242]	–1.17	0.310	0.821
ObjectVisual	+0.0065	+0.0188	–0.0122	[−0.0998, +0.0754]	–0.45	0.684	0.821
FaceVisual	–	–	–	–	–	–	–
SceneVisual	+0.0080	+0.0303	–0.0223	[−0.1590, +0.1144]	–0.64	0.582	0.821
MidTemporal	+0.0087	+0.0148	–0.0060	[−0.0392, +0.0272]	–0.51	0.638	0.821
EarlyAuditory	+0.0297	+0.0339	–0.0043	[−0.0538, +0.0452]	–0.24	0.822	0.897

The raw effect size is defined as Δ*r* = AVH+−AVH− and is reported with a two-sided Welch 95% confidence interval (CI). The *p* values are uncorrected; *q*_BH_ values were obtained using the Benjamini–Hochberg procedure across the 12 valid ROIs. No comparison survived FDR correction at *q*_BH_ < 0.05.

**Figure 3:**
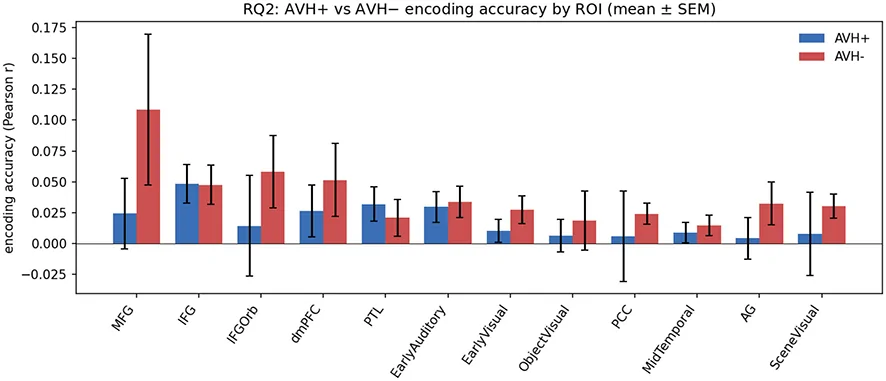
Encoding accuracy by ROI for AVH+ vs. AVH− (mean ± SEM).

This numerical pattern suggests that the SCZ>HC separation observed in RQ1 may be influenced more by the AVH− subgroup than by the AVH+ subgroup, although the AVH+–AVH− comparisons did not survive either nominal or FDR-corrected inference. In other words, AVH+ patients appear to track the HC group more closely, whereas AVH− patients show the strongest model–brain alignment. One possible interpretation is that auditory verbal hallucinations introduce internally generated speech-like activity that is not time-locked to the external auditory stimulus. Such internally generated activity would add variance to the BOLD signal that the stimulus-based encoding model cannot explain, thereby reducing encoding accuracy in AVH+ patients. However, this explanation remains speculative because the subgroup sample is extremely small and the effects are not statistically reliable. The result is best treated as a hypothesis-generating pattern: hallucination status may modulate speech-encoding alignment, but larger samples and trial-level hallucination measures are required to test this directly.

### 6.5 RQ3 finding: stimulus-condition effects are present, but the current feature space captures block-level stimulus presence more than speech meaning

To assess whether encoding alignment depends on the type of auditory stimulus, we compute language-ROI encoding accuracy separately for white-noise, words, sentences, and reversed-speech conditions across HC, AVH−, and AVH+ groups.

#### 6.5.1 Results

The condition-wise analysis revealed a group-dependent pattern in language ROIs (Table 9; Figure 4). The intelligibility contrast, defined as {words, sentences} minus {reversed, white-noise}, was negative in HC (−0.032) but slightly positive in SCZ (+0.007). This means that, in the current analysis, HC participants showed better model fit during low-level or unintelligible auditory blocks than during intelligible speech blocks. By contrast, SCZ participants showed a weak tendency toward higher alignment for intelligible speech. Among the schizophrenia subgroups, AVH− showed the strongest intelligible-speech alignment, with positive values for both words and sentences, whereas AVH+ showed weaker and more condition-specific responses.

**Table 9:** RQ3: Mean encoding accuracy across language ROIs, by group × condition.

Group	white-noise	words	sentences	reversed
HC	+0.0436	–0.0155	–0.0228	–0.0171
AVH-	–0.0046	+0.0196	+0.0133	+0.0269
AVH+	+0.0233	–0.0036	+0.0084	–0.0343

**Figure 4:**
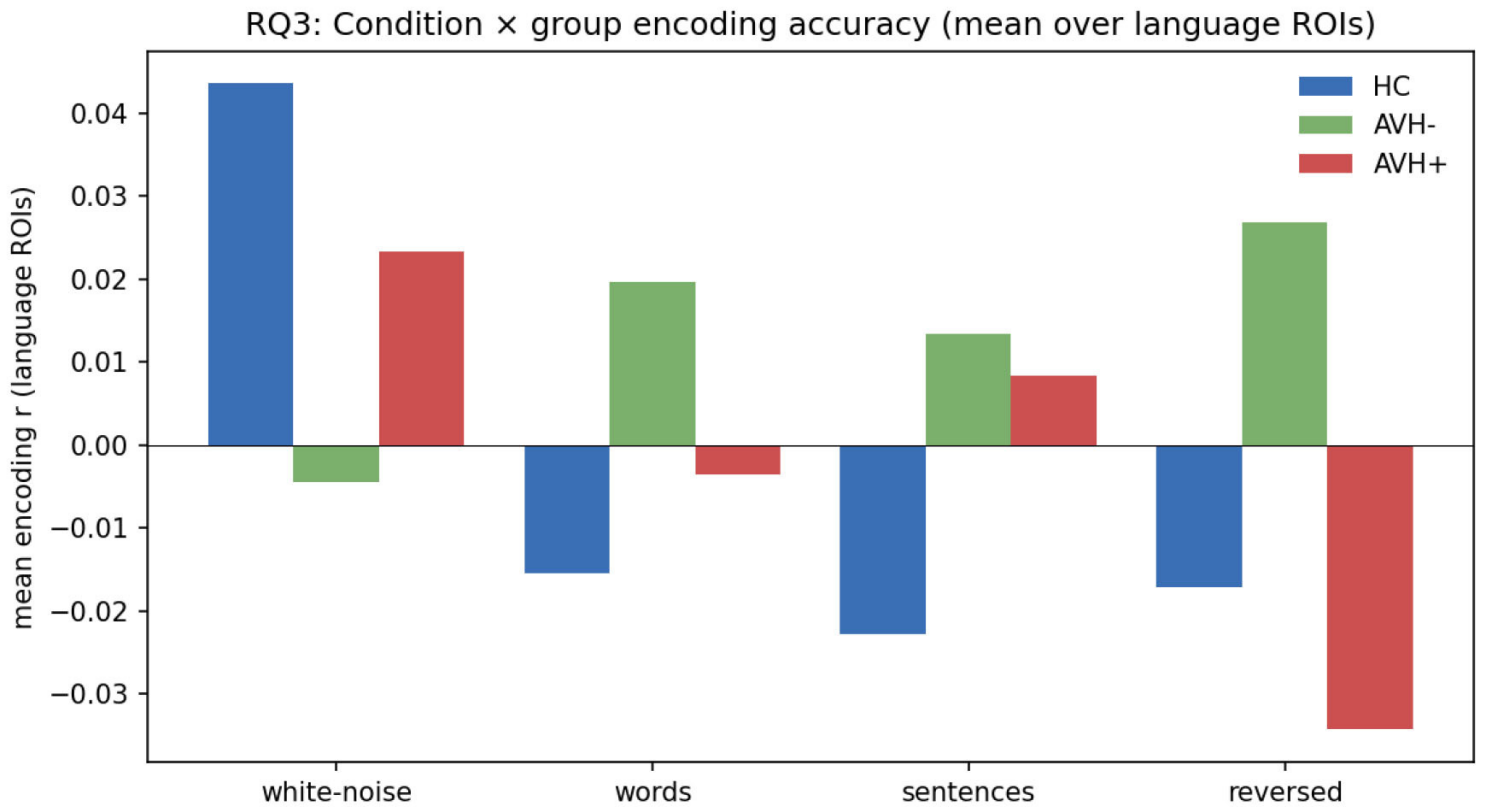
Mean encoding accuracy across the 8 language ROIs, by stimulus condition and group. Per-condition *r* uses only test TRs falling within blocks of that condition (HRF-shifted by 3 TRs).

The largest HC vs. SCZ difference occurred for sentences (*t* = −1.83, *p* = 0.116 uncorrected), again following the direction SCZ>HC. However, the interpretation of this result is limited by the minimal feature representation used in the current model. Because the feature space is relatively coarse, condition-specific encoding accuracy may reflect block-level stimulus presence, acoustic energy, or condition-specific variance rather than true semantic or linguistic representation. The negative intelligibility effect in HC especially suggests that the model may not yet be capturing meaning-specific structure in a reliable way. A stronger test of RQ3 would require richer acoustic, phonological, lexical, and semantic regressors, ideally separated into hierarchical feature sets. This would allow future analyses to determine whether group differences arise from low-level auditory processing, intelligibility, semantic integration, or internally generated speech-related activity.

### 6.6 RQ4 finding: frontal language regions carry the strongest encoding signal and the largest group differences

To identify which brain regions contribute most strongly to the encoding model and whether group effects are regionally specific, we rank ROIs by pooled encoding accuracy and examine HC–SCZ differences across regions.

#### 6.6.1 Results

The regional-specificity analysis showed that the strongest pooled encoding accuracy occurred in frontal language and control-related regions rather than in posterior temporal or sensory regions (Table 10; Figure 5). The highest pooled accuracies were observed in MFG (*r* = +0.043), IFG (*r* = +0.038), IFGOrb (*r* = +0.026), and dmPFC (*r* = +0.024), followed by PTL (*r* = +0.021) and EarlyAuditory (*r* = +0.017). In contrast, posterior language regions such as AG and PCC, as well as visual control ROIs, showed weaker pooled alignment.

**Table 10:** RQ4: ROIs ranked by pooled (across-group) mean encoding accuracy.

ROI	HC	AVH−	AVH+	Pooled	SEM	95% CI
**MFG**	–0.0032	+0.1085	+0.0242	+0.0432	0.0336	[−0.1014, +0.1878]
**IFG**	+0.0193	+0.0476	+0.0483	+0.0384	0.0096	[−0.0027, +0.0795]
**IFGOrb**	+0.0045	+0.0581	+0.0143	+0.0256	0.0165	[−0.0453, +0.0965]
dmPFC	–0.0055	+0.0516	+0.0266	+0.0242	0.0165	[−0.0469, +0.0953]
**PTL**	+0.0111	+0.0208	+0.0320	+0.0213	0.0060	[−0.0047, +0.0473]
EarlyAuditory	–0.0116	+0.0339	+0.0297	+0.0173	0.0145	[−0.0451, +0.0798]
EarlyVisual	+0.0128	+0.0273	+0.0103	+0.0168	0.0053	[−0.0060, +0.0396]
ObjectVisual	+0.0058	+0.0188	+0.0065	+0.0104	0.0042	[−0.0078, +0.0285]
PCC	–0.0018	+0.0241	+0.0061	+0.0095	0.0077	[−0.0235, +0.0424]
MidTemporal	+0.0002	+0.0148	+0.0087	+0.0079	0.0042	[−0.0103, +0.0261]
**AG**	–0.0168	+0.0324	+0.0043	+0.0066	0.0143	[−0.0547, +0.0679]
SceneVisual	–0.0272	+0.0303	+0.0080	+0.0037	0.0167	[−0.0683, +0.0757]

SEM and 95% CIs quantify variation across the three group means (HC, AVH−, and AVH+;). Boldface denotes canonical language-network ROIs.

**Figure 5:**
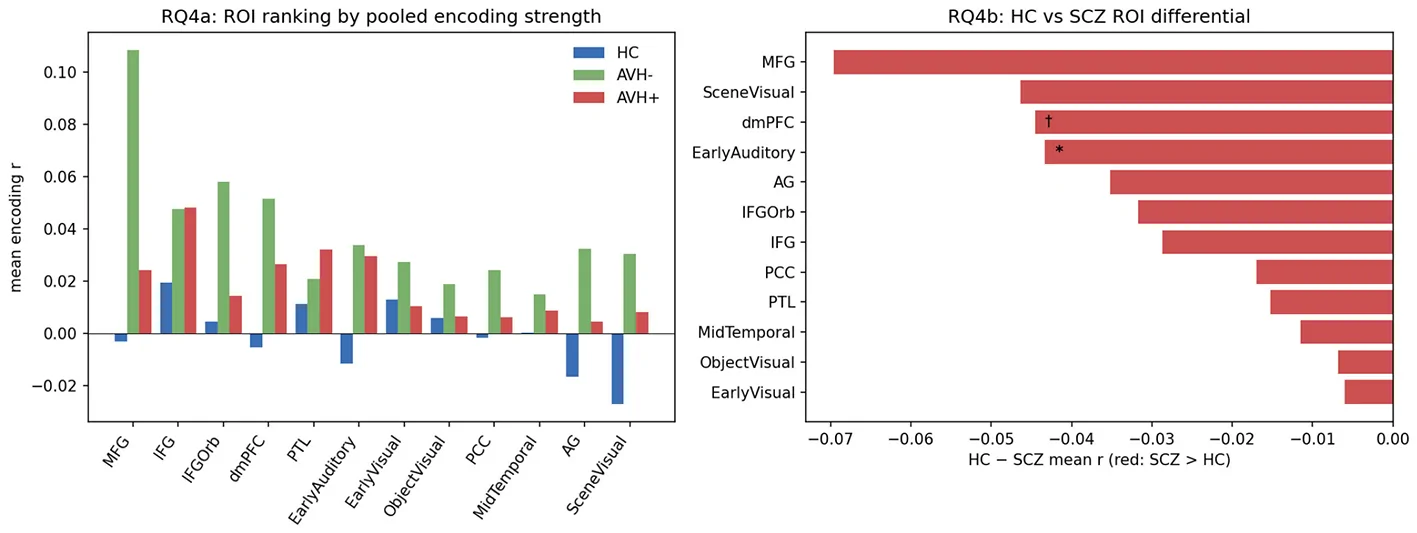
**(Left)** Per-ROI encoding accuracy by group, ROIs ordered by pooled strength. **(Right)** HC−SCZ differential per ROI (red: SCZ>HC). ^*^p < 0.05, ^†^*p* < 0.10, both uncorrected.

This pattern suggests that the model is not only capturing low-level auditory responses, but may also be sensitive to higher-order speech, semantic, and executive components represented in frontal language regions. Importantly, the largest HC vs. SCZ differences also appeared in these same frontal regions and in EarlyAuditory cortex. This co-localization indicates that the group effect is not uniformly distributed across the brain; instead, it is concentrated in regions plausibly involved in speech comprehension, cognitive control, and self/other-source attribution. Such a pattern is consistent with theories of schizophrenia that emphasize altered frontotemporal coordination and abnormal monitoring of internally vs. externally generated speech.

At the same time, the regional interpretation remains preliminary. The ATL ROI was excluded because of the medial-wall mask, leaving out a key anterior temporal language region that is often important for semantic representation. Future analyses should recover ATL using per-subject ROI masking or adjusted surface/volume alignment. In addition, the current ranking should be tested with stronger statistical controls, subject-level reliability estimates, and feature-space comparisons. Overall, RQ4 suggests that frontal language nodes are the most informative regions in the current encoding model, but the conclusion should be framed as an emerging regional pattern rather than a definitive localization result.

## 7 Discussion

This study applied voxelwise brain encoding models to a publicly available speech-perception fMRI dataset comprising schizophrenia patients with and without auditory verbal hallucinations and matched healthy controls (Soler-Vidal et al., 2022). By using self-supervised speech-model representations as the encoding feature space, we asked whether and where model–brain alignment differs between groups, whether it is modulated by hallucination status, how it varies across stimulus conditions, and which regions carry the strongest signal. We treat the four analyses as a coherent descriptive pattern rather than a confirmatory test, and discuss their interpretation, the limitations that constrain them, and the directions they suggest.

### 7.1 Principal findings

We emphasize at the outset that the clinical group results are exploratory. Although no HC–SCZ or AVH+–AVH− ROI comparison survived FDR correction, the pooled feature-family and baseline analyses produced corrected evidence that the encoding model captures information beyond simple task structure. Specifically, the complete model exceeded the rich non-deep baseline in 7 of 12 ROIs, while Wav2Vec 2.0 and HuBERT produced significant unique increments in 5 and 4 ROIs, respectively. Thus, the supported contribution comprises both a methodological demonstration and evidence for intermediate speech-model contributions across the pooled sample; it does not establish the direction or clinical utility of a schizophrenia-related group effect.

Across all evaluated ROIs, schizophrenia patients showed numerically higher encoding alignment than controls, with the largest numerical differences occurring in frontal language regions and EarlyAuditory cortex; AVH− participants also showed numerically higher alignment than AVH+ participants in most ROIs, although no group or subgroup comparison survived FDR correction. The effects were small in absolute terms (per-ROI (r) differences (≤0.07)), although their direction was consistent across the 12 valid ROIs. The largest numerical differences occurred in MFG, dmPFC, Scene Visual, and Early Auditory. However, because none survived correction and no corrected group-by-ROI effect was demonstrated, these observations do not establish that the group difference is regionally localized rather than reflecting a broader sample-level offset.

The frontal regions with the largest numerical HC–SCZ differences also showed relatively strong pooled encoding accuracy. This numerical overlap motivates regional hypotheses for future work but does not, by itself, demonstrate co-localization of a schizophrenia-specific effect. Within the schizophrenia group, AVH− participants showed numerically higher alignment than AVH+ participants in most ROIs, but every subgroup confidence interval included zero and no comparison survived FDR correction. The present data therefore do not establish that the AVH− subgroup drove the HC–SCZ pattern.

Condition-wise analyses likewise showed numerical group differences without corrected evidence for a group-by-condition effect. The baseline analyses demonstrated that the complete model predicts held-out neural responses beyond block timing, condition identity, acoustic, intelligibility, and simple lexical controls. Nevertheless, the absence of a significant unique BERT increment limits conclusions about meaning-specific or lexical-semantic representation in this controlled block-design paradigm.

### 7.2 Interpretation: a counterintuitive direction

The direction of the group difference, higher encoding alignment in schizophrenia, runs against the intuition that disorders disrupting auditory-linguistic representation should reduce stimulus-locked model fit. Several non-exclusive explanations deserve consideration. One possibility is statistical: with small per-ROI effects and weak overall signal in some subjects, group means are sensitive to individual variability, and the consistent direction may reflect a residual sample-level offset rather than a true representational gain. The outlier-sensitivity check on the HC group is consistent with this reading. A second possibility is methodological: encoding accuracy is computed against a relatively coarse, pooled feature space, and patients may show altered but more model-consistent responses in some regions for reasons not specific to speech processing (e.g., reduced task-unrelated variance, attentional narrowing, or differences in head motion-related noise structure). A third possibility, more speculative, is representational: predictive-coding accounts of psychosis (Adams et al., 2013; Sterzer et al., 2018; Corlett et al., 2019) posit overly precise top-down priors in schizophrenia, which could in principle yield stronger, more stereotyped stimulus-locked activity in frontal regions that the encoding model captures. We emphasize that the present data cannot distinguish these accounts; they are listed to indicate that a counterintuitive direction is not by itself disqualifying, but it does require richer features and replication before any representational interpretation is warranted.

The AVH-status pattern, while not statistically reliable, is internally consistent with a mechanistic reading often advanced in the AVH literature: that endogenously generated speech-like activity in hallucination-prone patients adds variance to the BOLD signal that a purely stimulus-driven encoding model cannot explain, lowering measured alignment (Ćurčić-Blake et al., 2017; Jardri et al., 2011). Under this view, AVH− patients would be expected to show the cleanest stimulus-locked alignment among the patient subgroups, which matches the observed pattern. We stress that this interpretation is hypothesis-generating; trial-level or state-based hallucination measures would be required to test it directly, since the present design contrasts trait AVH status during external speech, not active hallucination episodes.

### 7.3 Relation to prior work

Our regional pattern is consistent with the broader schizophrenia literature in two respects. The early-auditory effect aligns with reports of attenuated primary auditory cortex responses and altered low-level auditory tracking in patients (Javitt and Sweet, 2015; Sweet et al., 2009; Dondé et al., 2024; Soler-Vidal et al., 2022), including in the dataset re-analyzed here. The frontal (MFG, dmPFC, IFG/IFGOrb) concentration of group differences is consistent with theories that emphasize altered frontotemporal coordination and abnormal monitoring of internally vs. externally generated speech (Ćurčić-Blake et al., 2017; Sterzer et al., 2018). Methodologically, this work extends voxelwise encoding from the neurotypical setting (Schrimpf et al., 2021; Vaidya et al., 2022; LeBel et al., 2021; Antonello et al., 2024) to a clinical contrast that, to our knowledge, has not previously been examined with self-supervised speech-model features, complementing recent encoding-style applications in adjacent psychiatric contexts (Moussa et al., 2025; Dondé et al., 2024). The fact that the original *Soler-Vidal* analysis (Soler-Vidal et al., 2022) found no significant univariate AVH+ vs. AVH− differences on this task, whereas our model-based readout reveals a directionally consistent numerical separation, supports the broader argument that encoding-model alignment may be a more sensitive index than condition-contrast activation, even if our particular implementation is not yet statistically reliable.

### 7.4 Limitations

Several limitations bound the conclusions we can draw. First, although the dataset comprises 71 participants across three groups, the per-group effect magnitudes are small and none of the clinical group ROI-wise differences survive multiple-comparison correction; the clinical findings are descriptive, not confirmatory. Second, the feature-family contributions were separated using drop-family comparisons within the banded-ridge framework (Nunez-Elizalde et al., 2019; La Tour et al., 2022). These pooled analyses identified significant unique contributions from acoustic, Wav2Vec 2.0, and HuBERT features across distinct ROI sets, while BERT did not produce a significant unique increment in any ROI. The baseline analyses further showed that the complete model improved held-out prediction beyond block timing, task structure, acoustic, intelligibility, and simple lexical controls. However, because the feature-family analyses were conducted across the pooled sample, they do not establish that acoustic, speech-model, or linguistic representations are differentially disrupted in schizophrenia or by hallucination status; that conclusion would require corrected group-by-feature-family comparisons. Third, the ATL ROI was excluded due to the medial-wall mask, removing a region of known semantic relevance; future work should recover ATL via per-subject ROI definition. Fourth, the sub-03 outlier observation indicates that individual-subject variability disproportionately influences group means at this sample size, and the analyses would benefit from explicit influence diagnostics and pre-registered exclusion criteria. Fifth, the AVH+ vs. AVH− contrast captures trait, not state, hallucination status; encoding analyses with concurrent hallucination reports would more directly test the internally-generated-activity account. Finally, we have not analyzed the relationship between encoding accuracy and PANSS positive-symptom severity; such an analysis is a natural extension but lies outside the present descriptive scope.

### 7.5 Future directions

We propose a concrete three-step follow-up plan. (1) Hierarchical feature decomposition: separate low-level acoustic descriptors (log-mel, MFCCs), intermediate self-supervised speech representations (wav2vec 2.0, HuBERT), and high-level contextual linguistic embeddings into distinct bands within a banded ridge framework with variance partitioning, so that level-specific contributions can be estimated rather than pooled. (2) Level-specific regional tests: test whether acoustic, speech-model, and linguistic bands differ between groups specifically in early auditory cortex and in frontal language regions (MFG, dmPFC, IFG), the regions that carried both the strongest signal and the largest group differences here. (3) Symptom linkage in a pre-registered larger cohort: in an independent, adequately powered sample, relate level-specific encoding accuracy to PANSS positive-symptom scores and to hallucination frequency/state measures, with pre-registered ROIs, exclusion criteria, and correction procedures. Steps (1) and (2) can be executed on the present dataset; step (3) requires new data.

The most direct extensions follow from the limitations above. A hierarchical feature decomposition, separating low-level acoustic descriptors, intermediate self-supervised speech representations, and high-level contextual linguistic embeddings within a banded ridge framework, would allow the field's central representational question (which level is most disrupted, and where?) to be answered with the present dataset. Pre-registered replication in an independent and larger cohort would convert the current descriptive pattern into a confirmatory test. Linking encoding accuracy to symptom-severity measures (PANSS positive scores, hallucination frequency, source-monitoring task performance) would address the clinical-relevance question that motivates the broader biomarker framing. Methodologically, individual-subject reliability ceilings, subject-level outlier diagnostics, and trial-level state measures of hallucination would substantially strengthen any future SCZ-vs-HC encoding contrast. More broadly, model-based encoding has so far been validated almost entirely in neurotypical participants; demonstrating that the framework yields reproducible, clinically interpretable contrasts in psychiatric populations remains an open and worthwhile goal.

### 7.6 Conclusion

We presented a voxelwise encoding-model analysis of speech perception in schizophrenia, using self-supervised speech-model representations to quantify model–brain alignment in patients with and without auditory verbal hallucinations and in matched controls. Across all evaluated ROIs, schizophrenia patients showed numerically higher encoding alignment than controls, with the largest differences concentrated in frontal language regions and early auditory cortex, and with the non-hallucinating subgroup driving most of the numerical separation. The effects are small and do not survive multiple-comparison correction; we therefore present them as a descriptive, hypothesis-generating pattern rather than as evidence of preserved or enhanced speech encoding in schizophrenia. The principal contribution is methodological and representational: the analysis demonstrates that computational encoding models can be applied to a clinical speech-perception fMRI dataset and that intermediate speech-model features improve held-out prediction beyond the measured non-deep baselines, while the clinical group patterns require larger-sample confirmation.

## Data Availability

The data analyzed in this study are publicly available in OpenNeuro under accession ds004302 at https://doi.org/10.18112/openneuro.ds004302.v1.0.0. The original contributions presented in the study are included in the article; further inquiries can be directed to the corresponding author.
